# Evaluation of treatment response and resistance in metastatic renal cell cancer (mRCC) using integrated ^18^F–Fluorodeoxyglucose (^18^F–FDG) positron emission tomography/magnetic resonance imaging (PET/MRI); The REMAP study

**DOI:** 10.1186/s12885-017-3371-9

**Published:** 2017-06-02

**Authors:** Christian Kelly-Morland, Sarah Rudman, Paul Nathan, Susan Mallett, Giovanni Montana, Gary Cook, Vicky Goh

**Affiliations:** 1 Department of Cancer Imaging, King’s College London Division of Imaging Sciences & Biomedical Engineering, St Thomas’ Hospital, Westminster Bridge Road, London, SE1 7EH UK; 2 Department of Medical Oncology, Guy’s Hospital, Great Maze Pond, London, SE1 9RT UK; 3 Department of Medical Oncology, Mount Vernon Cancer Centre, Rickmansworth Road, Northwood, Middlesex, HA6 2RN UK; 4 Birmingham Clinical Trials Unit, Institute of Applied Health Research, University of Birmingham, B15 2TT, Birmingham, UK; 5 Department of Biomedical Engineering, King’s College London Division of Imaging Sciences & Biomedical Engineering, St Thomas’ Hospital, Westminster Bridge Road, London, SE1 7EH UK

**Keywords:** Metastatic renal cell carcinoma, Magnetic resonance imaging, Positron emission tomography, Pet/MRI, Response assessment

## Abstract

**Background:**

Tyrosine kinase inhibitors are the first line standard of care for treatment of metastatic renal cell carcinoma (RCC). Accurate response assessment in the setting of antiangiogenic therapies remains suboptimal as standard size-related response criteria do not necessarily accurately reflect clinical benefit, as they may be less pronounced or occur later in therapy than devascularisation. The challenge for imaging is providing timely assessment of disease status allowing therapies to be tailored to ensure ongoing clinical benefit. We propose that combined assessment of morphological, physiological and metabolic imaging parameters using 18F–fluorodeoxyglucose positron emission tomography/magnetic resonance imaging (^18^F–FDG PET/MRI) will better reflect disease behaviour, improving assessment of response/non-response/relapse.

**Methods/design:**

The REMAP study is a single-centre prospective observational study. Eligible patients with metastatic renal cell carcinoma, planned for systemic therapy, with at least 2 lesions will undergo an integrated ^18^F–FDG PET and MRI whole body imaging with diffusion weighted and contrast-enhanced multiphasic as well as standard anatomical MRI sequences at baseline, 12 weeks and 24 weeks of systemic therapy allowing ^18^F–FDG standardised uptake value (SUV), apparent diffusion co-efficient (ADC) and normalised signal intensity (SI) parameters to be obtained. Standard of care contrast-enhanced computed tomography CT scans will be performed at equivalent time-points. CT response categorisation will be performed using RECIST 1.1 and alternative (modified)Choi and MASS criteria. The reference standard for disease status will be by consensus panel taking into account clinical, biochemical and conventional imaging parameters. Intra- and inter-tumoural heterogeneity in vascular, diffusion and metabolic response/non-response will be assessed by image texture analysis. Imaging will also inform the development of computational methods for automated disease status categorisation.

**Discussion:**

The REMAP study will demonstrate the ability of integrated ^18^F–FDG PET-MRI to provide a more personalised approach to therapy. We suggest that ^18^F–FDG PET/MRI will provide superior sensitivity and specificity in early response/non-response categorisation when compared to standard CT (using RECIST 1.1 and alternative (modified)Choi or MASS criteria) thus facilitating more timely and better informed treatment decisions.

**Trial registration:**

The trial is approved by the Southeast London Research Ethics Committee reference 16/LO/1499 and registered on the NIHR clinical research network portfolio ISRCTN12114913.

## Background

Renal cell carcinoma (RCC) is currently the ninth commonest cancer with a worldwide incidence of 2.4% in 2012 [1]. Usually presenting in the sixth and seventh decades of life, and commoner in men than women, the incidence of the disease has been rising steadily at approximately 1.1% per year over the last decade [2]. The commonest histological subtype of RCC is clear cell, accounting for 70% of cases with papillary RCC and the more indolent chromophobe subtype contributing to the remaining majority. Rarer entities such as the aggressive collecting duct carcinoma account for less than 5% of cases [3]. The five-year survival rate in metastatic RCC remains poor at less than 12% [4]. Metastatic disease is present in up to 30% of patients at the time of diagnosis with up to a further 30% of patients subsequently developing metastases in the ensuing two years despite resection of the primary tumour [5].

### Treatment options

Systemic treatment options for metastatic RCC have shifted to targeted therapies in the last decade following demonstration of improved progression free survival in Phase III clinical trials [6–12]. Renal cell carcinoma can be a highly vascular tumour, for example, related to Von-Hippel Lindau gene mutations and accumulation of hypoxia inducible factor (HIF). Transcriptional targets of HIF include vascular endothelial growth factor (VEGF) and platelet derived growth factor (PDGF), with upregulation of angiogenesis, hence why anti-angiogenic targeted therapies have been successful in metastatic RCC.

**Fig. 1:**
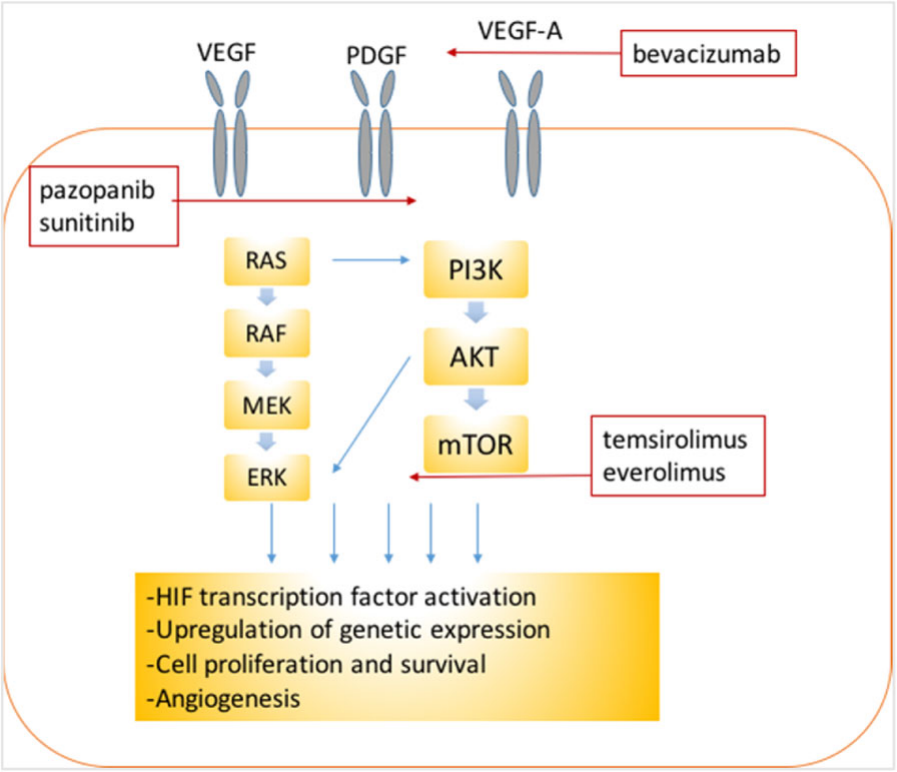
Illustrating the sites of action of the targeted chemotherapies in renal cell carcinoma. The cell proliferation and angiogenisis cell-signalling pathways are up-regulated in malignancy and can be targeted at the level of the receptors (Pazopinib, Sunitinib), agonist (Bevacizumab) or downstream signalling pathways (Temsirolimus, Everolimus). VEGF: Vascular endothelial growth factor, PDGF: Platelet derived growth factor, mTOR: mammalian target of rapamycin, PI3K: phosphatidylinositol 3-kinase, AKT: protein kinase B, ERK: Extracellular signal-regulated kinase

A number of targeted agents have received FDA approval for metastatic RCC. These include the small molecule multi-kinase inhibitors including pazopinib, sunitinib, sorafenib and axitinib, the mammalian target of rapamycin (mTOR) inhibitors everolimus and temsirolimus, and the monoclonal antibody Bevacizumab, an inhibitor of vascular endothelial growth factor A. These agents selectively inhibit neo-angiogenesis and cell proliferation respectively through a variety of signalling pathways [13] including the RAS-RAF-MEK-ERK and PI3K-AKT-mTOR pathways (Fig. 1). These agents have been shown to positively impact survival with transient stabilisation of disease in 70% of patients as well as providing scope for several lines of treatment [6–12]. More recently clinical trials of immunotherapeutic agents have also shown promise. These agents stimulate the immune system to destroy cancer cells and the possibility of a synergistic effect between immunotherapy and targeted therapy is under investigation [4]. The CheckMate 025 phase III multicentre trial of 821 patients demonstrated that the programmed (T-cell) death-1 (PD-1) inhibitor, nivolumab, which potentiates the native T-cell response has demonstrated favourable an overall survival benefit with a 27% reduction in the risk of death as well as a greater response rate (25% versus 5%) when compared to treatment with everolimus [14].

### The need for alternative imaging response biomarkers

Our preliminary work in patients treated with multi-kinase inhibitors has shown that different clinical imaging patterns may be observed with treatment [15]. New metastases may appear even if pre-existing lesions remain stable. Changes in size and/or vascularisation of existing lesions may occur either rapidly or slowly. Proposed vascular mechanisms of resistance include: 1) Up-regulation of alternative pro-angiogenic pathways, allowing the development of new abnormal tumour vessels, restoring tumour angiogenesis to its pre-treatment level and allowing rapid tumour growth; 2) Development of alternative metabolic pathways independent of vascular supply, which may represent a more aggressive phenotype; 3) Recruitment of vascular progenitor cells, pro-angiogenic monocytes, pericytes, or co-option of normal vessels, thus reconstituting vessels which do not have the typical characteristics of tumour vessels. These pseudo-normal vessels are inefficient resulting in a slower tumour re-growth rate.

Assessing response to targeted therapies with the internationally accepted response evaluation criteria in solid tumours version 1.1 (RECIST 1.1) has been challenging in this setting. With RECIST 1.1 percentage changes in the sum of maximal unidirectional measurements of target visceral or short axis nodal metastatic lesions over time are used to categorise treatment response, disease stability or progression [16]. However targeted therapies do not necessarily lead to a concomitant size reduction [15, 17]. Indeed immunotherapies such as ipilimumab and pembrolizumab may also initially increase lesion size, in up to 15% of cases during successful therapy (‘pseudoprogression’) [18]. This may be due to the accumulation of inflammatory infiltrates secondary to the augmented immune response [19]. One such study of Pembrolizumab in 411 patients treated for metastatic melanoma found that 12% of patients demonstrated an initial increase in metastatic lesions that would have led to a classification of progressive disease by RECIST 1.1 despite a decline in overall tumour burden over time [20, 21]. In order to prevent premature cessation of efficacious treatment and conversely continued treatment with ineffective treatment there is a need to improve imaging response assessment. An example of preliminary work is the incorporation of changes in tumour density (Hounsfield unit attenuation) measured on contrast enhanced computed tomography (CT) with size change [22, 23] and immune response criteria [20].

We propose that in vivo imaging targeted at the mode of drug action, its downstream effects, and a better understanding of the mechanisms underlying tumour response and resistance will enable imaging to provide a better indication of therapeutic effect. Hybrid positron emission tomography/magnetic resonance imaging (PET/MRI) scanners enables PET tracers such as the glucose analogue fluorine 18 (^18^F) fluorodeoxyglucose (FDG) to be combined with multi-parametric MRI including multiphasic contrast enhanced and diffusion weighted sequences. Preliminary studies of ^18^F–FDG PET alone [24–30] or MRI alone [31–34] have suggested a role for these modalities as response biomarkers. ^18^F–FDG PET has a high sensitivity and specificity for extra-renal lesions with a sensitivity and specificity of up to 84% and 91% in detecting metastases, which appear hypermetabolic and and a Phase II study of 44 patients has shown that reduction in the FDG metabolic activity and tumour size is associated with overall and progression free survival [24]. A high baseline FDG standardised uptake value (SUV) also correlates with disease aggressiveness [26, 35, 36].

The REMAP study will examine whether the performance of integrated FDG-PET/MRI is better than standard of care imaging for response categorisation in metastatic RCC patients undergoing targeted therapy. We hypothesise that integrated ^18^F–FDG PET/MRI combining anatomic, metabolic and physiological imaging will be able to detect response/resistance ahead of size change allowing non-responding patients to transition to alternative therapies more swiftly. An integrated whole body technique will also allow for the heterogeneity of response to be explored within and between lesions, building on existing work [37]. PET/MRI will also facilitate the assessment of potential effects of therapy e.g. angiogenesis inhibition or immune modulation on normal organ microvasculature, which underlie treatment co-morbidities, e.g. hypertension as suggested in our previous work [38].

## Methods/design

### Study design

The REMAP study is an investigator initiated single centre observational study. All participants will have to provide written informed consent, signed and dated before inclusion in the study. The study is registered on the NIHR CRN portfolio ISRCTN12114913.

### Study organisation

The sponsor is King’s College London and Guy’s & St Thomas’ Hospitals NHS Trust, London. The study is funded by Cancer Research UK (CRUK award reference C42827/A20000) and will be carried out at the King’s College London and Guy’s and St Thomas’ PET Centre, London.

### Ethical approval

The final protocol has been approved by the Southeast London Research Ethics Committee (ref: 16/LO/1499) and approved by the Administration of Radioactive Substances Advisory Committee of the Dapratment of Health (ARSAC), UK.

### Study population

Thirty eight patients with proven metastatic renal cell carcinoma will be recruited into the study. Patient inclusion and exclusion criteria are summarised in Table 1. A staging CT is required. Study entry will be restricted to patients in whom there is at least one lesion 2 cm or greater in size in order to minimise partial volume effects in ^18^F–FDG PET/MRI.

**Table 1:** Patient recruitment

Inclusion criteria	
1. Adults (>18 years) capable of providing informed consent with metastatic renal cell cancer	
2. Eastern cooperative oncology group (ECOG) performance status ≤ 2	
Exclusion criteria	
1. Estimated prognosis < 12 weeks	
2. Contraindications to contrast enhanced CT, MRI or FDG/PET	
3. Women who are pregnant or lactating	

### Study objectives & endpoints

The primary objective of the REMAP study is to compare integrated ^18^F–FDG PET/MRI with CT for response categorisation at 12 weeks and 24 weeks on treatment and to assess the sensitivity and specificity of integrated ^18^F–FDG PET/MRI metrics to predict for disease progression after 12 and 24 weeks on treatment (PET: FDG standardised uptake value (SUL_peak_); MRI: diffusion MRI apparent diffusion co-efficient (ADC) and post-gadolinium contrast agent normalised tumour signal intensity (SI_norm_), (alone or in combination).

Secondary objectives are; 1) to compare ^18^F–FDG PET/MRI response categorisation versus RECIST 1.1 and exploratory combined size & enhancement criteria (e.g. Choi, modified Choi and MASS [39–41]; 2) to assess if whole body tumour burden is better than target lesions only for response categorisation (RECIST 1.1); 3) to assess and quantify the inter-lesional heterogeneity in responding/non-responding lesions by texture analysis and 4) to explore whether there is a consistent effect of therapy on normal organ physiology e.g. kidney, liver, spleen.

The primary endpoint is the number of responders/non-responders on ^18^F–FDG PET/MRI versus standard CT at 12 and 24 weeks and a consensus reference standard based on all patient information up to 36 weeks. Time to progression and progression free survival beyond 36 weeks will also be recorded in order to determine if ^18^F–FDG PET/MRI metrics can predict for disease progression.

### Imaging

#### Computed tomography (CT)

Contrast enhanced CT of the thorax, abdomen and pelvis (100-120kVp, dose modulated mA, matrix 512 × 512, field of view 35 cm, ≤5 mm reconstructed slice thickness, following intravenous administration of 100 ml of ≥300 mg/ml iodinated contrast agent at 3 mL/s) will be performed as part of the patient’s standard clinical care at baseline and 12, 24 and 36 weeks ±21 days post-commencement of systemic therapy.

#### ^18^F FDG-pet/MRI

Up to 400 MBq of ^18^F–FDG will be administered intravenously. Following a 60 ± 5 min uptake period simultaneous ^18^F–FDG PET/MRI will be undertaken. ^18^F–FDG PET with a 2-point Dixon based segmentation technique utilised for attenuation correction, T1-weighted, T2-weighted, diffusion weighted (b-values: 50, 900 s/mm^2^) and multiphasic post contrast T1-weighted sequences (arterial, portal venous and equilibrium) following intravenous administration of gadolinium (0.1 mmol/kg) will be obtained from vertex to mid-thigh. ^18^F–FDG PET/MRI will be performed within 2 weeks of the standard CT scans. Each ^18^F–FDG PET/MRI will incur a maximal estimated radiation dose of 7.3mSV compared to a maximal estimated 19mSV per CT of the thorax, abdomen and pelvis.

### Categorisation of response

#### RECIST 1.1

Categorisation of response, disease stability or disease progression will be by RECIST 1.1 at 12, 24 and 36 weeks for target and non-target lesions. This is summarised in Table 2. This will be performed for each CT.

**Table 2:** Response assessment criteria

	RECIST	CHOI	mCHOI	MASS
Progressive disease (MASS unfavourable response)	Increase in sum of longest target lesion diameters >20%. or Development of new lesions. or Unequivocal non-target lesion progression.	Increase in lesion size^a^ > 10%. or Development of new lesions. or New or enlarging intratumoral nodule.	Increase in lesion size^a^ > 10%. or Development of new lesions. or New or enlarging intratumoral nodule.	Increase in lesion size^a^ > 20% without central necrosis. or Development of new lesions. or New intratumoral enhancing components.
Partial response (MASS favourable response)	>30% decrease in sum of longest target lesion diameters.	Decrease in target lesion CT enhancement (Hounsfield units) >15% *or* size >10%	Decrease in target lesion CT enhancement (Hounsfield units) >15% *and* size >10%	Decrease in lesion size >20%, central necrosis or reduction in attenuation >40 Hounsfield units
Complete response	Disappearance of all target lesions and resolution of lymphadenopathy (<10 mm).	Disappearance of all target lesions	Disappearance of all target lesions	Disappearance of all target lesions
Stable disease (MASS indeterminate response)	None of the above	None of the above	None of the above	None of the above

^a^Lesion size refers to the sum of the longest diameter of up to 10 target lesions (two per organ)

#### Size & enhancement criteria

Categorisation of response, disease stability or disease progression will be by Choi, modified Choi (mCHOI) and MASS criteria at 12, 24 and 36 weeks for target and non-target lesions. This is summarised in Table 2. This will be performed for each CT.

#### ^18^F FDG-PET/MRI metrics

Categorisation of response, disease stability or disease progression using ^18^F–FDG PET/MRI metrics are summarised in Table 3. For ^18^F–FDG PET alone this is as per PERCIST [42]. A measurable target lesion is defined as the single tumour lesion with highest uptake value. SUL_peak_ (maximal 1.2-cm diameter volume ROI in tumour) has to be at least 1.5-fold greater than liver SUL_mean_ in a 3-cm diameter ROI in normal right lobe of liver (or 2-fold greater than the SUL_mean_ of blood pool in 1-cm-diameter ROI in the descending thoracic aorta extended over 2-cm *z*-axis if the liver is abnormal).

**Table 3:** PET/MRI response categorisation

Category/Parameter	Complete Response (CR)	Partial Response (PR)	Stable Disease (SD)	Progressive Disease (PD)
^18^F FDG PET SUL_peak_	Resolution of target lesion activity: activity less than mean liver activity & indistinguishable from background blood-pool levels. Disappearance of all other lesions to background blood-pool levels.	≥30% reduction of target lesion SUL_peak._ Absolute decrease ≥0.8 SUL units. Measurement is commonly in same lesion as baseline but can be another lesion if this is now the most active lesion after treatment.	Does not fulfil criteria for response or progression	>30% increase of target lesion SUL_peak._ New ^18^F FDG avid lesions.
DW-MRI ADC_50–900_	Resolution of DWI signal	Persistence of DWI signal; >30% increase in ADC_mean_	Does not fulfil criteria for response or progression	Persistence of DWI signal; >25% reduction in ADC_mean_
DCE-MRI SI_normalised_	Resolution of enhancement	Persistence of enhancement; >40% decrease in SI_norm_	Does not fulfil criteria for response or progression	Persistence of enhancement; >40% increase in SI_norm_

#### Reference standard for response categorisation:

Disease status (PD/non-PD, per patient and per lesion) by consensus will be confirmed using clinical criteria and all conventional imaging up to 36 weeks on treatment. The time-point of PD up to 36 weeks defined by consensus will be recorded. This time point is based on previous data (Median TTP: 336 days [37]).

Additionally computational methods for predicting response/non-response will be developed using two complementary approaches: a) state-of-the-art machine learning models, e.g. boosting and support vector machines based on customised kernel functions; b) novel “deep” neural networks architectures that are capable of automatically extracting predictive features in a completely data-driven manner.

#### Sample size and analysis

This is a feasibility study with the sample size (*n* = 38) based on estimating parameters needed to design a future efficacy study. Parameters estimated from this feasibility study will be (i) the sensitivity and specificity of ^18^F–FDG PET/MRI (new test) to detect progressive disease after 12 & 24 weeks on treatment; (ii) the difference in sensitivity and correlation of ^18^F–FDG PET/MRI compared to CT RECIST (standard test). Additional assumptions tested will be the prevalence of disease progression and the best timepoint for detection of disease progression. The sample size of 38 patients, 19 with disease progression, will allow for an estimation of sensitivity of ^18^F–FDG PET/MRI with a 95% confidence intervals lower limit precision of within 20%. The expectation is that 95% of patients from the same clinical population will have a sensitivity within these 95% confidence intervals (CI). The assumptions for this feasibility study are a prevalence of patients with PD of 50%; ^18^F–FDG PET/MRI 80% sensitivity per patient for PD; and 10% loss to follow up. Sensitivity and specificity per patient will be compared using paired comparison of proportions of (i) ^18^F–FDG PET/MRI & (ii) CT RECIST to detect PD.

## Discussion

Accurate and timely response assessment in metastatic RCC is important to facilitate changes in treatment, in particular discontinuing non-efficacious, costly treatment that may be associated with toxicity. It is well known that conventional response assessment based on size does not best reflect the clinical effectiveness of cytostatic targeted therapies as size changes lag behind actual response and relapse in the majority of cases [15]. Assessment of the downstream effects of therapy on tumour vascularisation, glucose metabolism and water diffusion with integrated hybrid imaging potentially provides a more personalised approach to therapeutic evaluation.

It is well known that tumour vascularisation is heterogeneous. During growth tumours either co-opt a vascular supply from adjacent normal vessels, incite new vessel growth de novo (vasculogenesis), or from existing vasculature (angiogenesis) [43, 44]. The resultant tumour vessel architecture is disorganised and dysfunctional. Areas of adequate or hyperperfusion may co-exist with areas of hypoperfusion or high permeability (leading to increased intra-lesional interstitial fluid pressure and hypoxia). This variation in haemodynamic status will influence therapy delivery and its effectiveness [43]. Assessment of CT Hounsfield units (enhancement characteristics) in addition to size has already been performed to augment response assessment [15]. Several varying response patterns have been described in the literature ranging from a correlative reduction in tumour CT density (enhancement) and size to a reduction in tumour CT density with stabilisation of disease, or conversely minimal change in tumour CT density with at best disease stabilisation or progression [15].

There is little specific data of FDG PET for response assessment in metastatic RCC [45] but small studies (10–30 patients) have found that FDG PET metabolic response may occur within 1 cycle of therapy [46] and possibly as early as 2 weeks into treatment [47] with sunitinib or sorafenib as first or second line therapy.

There is also evidence of a strong correlation between the number and ^18^F–FDG avidity of lesions and overall survival in patients on targeted therapy [24]. One study examined 243 metastatic lesions in 26 patients and demonstrated a significantly worse median survival time amongst patients in whom the maximum SUV of the primary tumour was >8.8 [48]. A subsequent larger study involving 60 patients also showed a higher mortality of 62.5% in those patients in whom the maximum SUV of the primary was >10 versus 33.3% in cases where the maximum SUV measured 3–5.

Diffusion weighted MRI may also provide relevant biological information. Investigators have demonstrated that a positive correlation exists between primary tumour grade and ADC values in a group of 33 patients; there was a statistically significant downward trend in the ADC value of lesions as grade increased [49]. A further study of 47 patients with metastatic renal cell carcinoma did not find baseline whole tumour mean ADC to be a predictor of outcome prior to the commencement of targeted therapy but there was a positive correlation between overall survival and the proportion of tissue within the tumour below the 25th percentile point of the cumulative ADC histogram [33]. It has also been shown that diffusion weighted imaging and multiphasic contrast enhanced MRI signal intensity may alter in response to treatment in renal cell cancer [33].

There are few data correlating imaging with mechanisms of relapse. A number of potential mechanisms underlying varying responses have been proposed with preclinical studies demonstrating an up-regulation of the production of the pro-angiogenic cytokine IL-8 associated with the development of sunitinib resistance, the same study found that the tumours in murine models were subsequently re-sensitised to sunitinib following the administration of an IL-8 neutralising antibody and began to respond to therapy despite previous failure [50]. Another potential factor in the development of drug resistance is the upregulation of hypoxia inducible factor 1-alpha (HIF-1a) in response to the hypoxic microenvironment induced by anti-angiogenic therapy. The presence of HIF-1a can lead to upregulation of expression of the RTK MET genes leading to potentiation of the MAPK tumorigenesis pathways [51].

It is clear that further research is needed to add to the body of evidence supporting alternative imaging biomarkers of treatment response/non-response/relapse in this arena for which PET/MRI is ideally suited. Simultaneous acquisition of data relating to tumour metabolic activity, perfusion and diffusion in a single, streamlined, integrative examination will provide a more representative and comprehensive picture of underlying biological mechanisms of therapy. Data from this pilot study will also provide the necessary initial evidence to power a future multicentre study. This study will also explore the quantitation of heterogeneity of therapy response both within and between metastases and provide preliminary data for computational methods that are capable of automatically extracting predictive features in a completely data-driven manner.

## Conclusion

In conclusion, the REMAP study will provide initial evidence for the sensitivity and specificity of ^18^F–FDG PET/MRI to detect disease response/non-response in metastatic renal cell cancer treated with targeted therapies, and how its performance in response categorisation compares to standard CT RECIST 1.1.

## Abbreviation

ADC: Apparent diffusion coefficient
ARSAC: Administration of Radioactive Substances Advisory Committee
CR: Complete Response
CT: Computed Tomography
DCE: Dynamic contrast enhanced
DWI: Diffusion weighted imaging
DCE: Dynamic contrast enhancement
FDG: Fluorodeoxyglucose
GCP: Good Clinical Practice
Gd: Gadolinium
MRI: Magnetic Resonance Imaging
PD: Progressive Disease
PET: Positron Emission Tomography
PR: Partial Response
RECIST: Response evaluation criteria in solid tumours
rSI: Relative signal intensity
SD: Stable Disease
SUV / SUL: Standardised Uptake Value/Standardised Uptake value normalised to lean body mass
HIF-1a: Hypoxia inducible factor 1-alpha
RTK MET: Receptor tyrosine kinase MET
MAPK: Mitogen activated protein kinase
